# Supplementary material for: Learning curve and surgical outcome of robotic assisted colorectal surgery with ERAS program
Source: Sci Rep. 2022 Nov 29;12:20566. doi: 10.1038/s41598-022-24665-w (PMC9709162; doi:10.1038/s41598-022-24665-w)
Supplement: Supplementary file 2 — Supplementary Information 2. [file 41598_2022_24665_MOESM2_ESM.zip › RAL Raw data-Ñ[▒K/060/HS_ñw╝╨░O▒Kñσ.pdf]

正式摘要(電子簽章：000000210167283)		
W52 / W52 ☐英文標題(注意:無英文姓名-請補病人基本檔!)	☐【讀取】憑證	

臺中榮民總醫院
出院病歷摘要

轉入號 : 轉出號 :

[redacted]

(8)流水編號	
(9)入院日期	110/02/04 (2021) 直腸外科 W52 - 092
(10)轉科日期	
(11)出院日期	110/02/09 (2021) 住院天數計 5 日

診斷

- 入院 :Rectal cancer
- 出院 :Rectal cancer s/p robotic LAR with loop ileostomy on 2021/2/5

主訴

- For scheduled surgery

病史

- This 41 y/o female had past history of rectum cancer , who admitted for scheduled surgery.
  According to the patient, she suffered from bloody stool on 2020/11 and went to LMD for help. Pathology showed hemorroid. She was done colonoscopy at LMD on 2021/02/02 and found ulcerative tumor 4cm over rectum 7cm FAV. CT was also arranged and revealed rectal cancer with regional lymphadenopathy. For second opinion, she came to our OPD for help. Lab data showed elevated CEA, but CA199 wasn't elevated.
  After discussing in OPD, she was admitted for Robot Assisted Low Anterior Resection.

體檢發現

-
  - General appearance :
    - easy looking , conscious clear , without cardiopulmonary distress
    - Vital signs自動帶入date 體溫(BT) 脈搏(P) 呼吸(R) 血壓(BP) 身高(BH) 體重(BW) BMI
      20210204 36.6℃ 94次/分 20次/分 117/69mmHg 158.6cm 60.4kg 24.0
    - Vital signs: B.P. 117/69 mmHg , P.R. 94 /min , R.R. 20 /min , B.T. 36.6 ℃
  - HEENT :
    - Eyes: no anemic conjunctiva, no icteric sclera, normal EOM
    - ENT: no otorrhea, no rhinorrhea, no erythematous mucosa, no injected tonsil, no deformity
  - Neck: supple, freely movable, trachea midline,no deformity, no engorged jugular vein , no palpable thyroid or lymph node enlargement
  - Chest:
    - Inspection: Grossly normal, symmetric and fully expansion, no accessory muscle used, no operation scar, no spider angiomata, no gynecomastia
    - Palpation: no palpable mass or nodule, no subcutaneous emphysema, no tenderness
    - Ausculation: clear breathing sound
  - Heart:
    - Percussion: normal heart size
    - Ausculation: regular heart beat, normal S1, S2, no gallop, no murmur
  - Abdomen:
    - Inspection: in
      - flat contour, symetrically, no scar, no stria, no dilated veins, no rashes
    - Ausculation: normoactive bowel sound, no abdominal bruits
    - palpation: soft, no muscle guarding or rigidity, no local tenderness, no rebounding pain, no palpable mass, no palpable pulsation

-
  - Percussion: no shifting dulllness, no hepatomegaly, no splenomagaly
-

Peripheral pulsation :	CA	BA	RA	FA	PA	PTA	DPA
Right side :	++	++	++	++	++	++	++/+++
Left side :	++	++	++	++	++	++	++/+++

- Genito-anus: no tenderness or palpable mass lesion or rectal shelf, normal external genitalia, normal anal tone, brown stool

CRS CONDITION

-
  - Lung

手術日期及方法

- 手術日期及方法20210205 Robot Assisted Low Anterior Resection + loop ileostomy

住院治療經過

- After admission, ERAS protocal was started. Robotic LAR with loop ileostomy was done on 2/5. Foley was remove on 2/6 and started liquid diet. The patient had fair appetite and stool passage over ileostomy. Under stable condition, the patient was discharge with OPD follow up.

合併症

一般檢查

特殊檢查

病理報告

其它

出院時情況

- 3.改門診治療

出院指示

藥名	劑量	劑量單位	用法	頻次	數量	數量單位	現況	天數	單位
Ultracet tab	1	TAB	PO	Q8HPRN	21	TAB	TH	7	天
Panho tab 40mg	1	TAB	PO	QDAC	7	TAB	TH	7	天

出院計劃

主治醫師 2062F 蔣鋒帆 / 住院醫師 2332G 林志安

正式摘要(電子簽章：**000000210167283)**

W52 / W52

doctorRank:B
